# How Does Personal Hygiene Influence Indoor Air Quality?

**DOI:** 10.1021/acs.est.4c01698

**Published:** 2024-05-23

**Authors:** Nijing Wang, Tatjana Müller, Lisa Ernle, Gabriel Bekö, Pawel Wargocki, Jonathan Williams

**Affiliations:** † Atmospheric Chemistry Department, Max Planck Institute for Chemistry, 55128 Mainz, Germany; ‡ International Centre for Indoor Environment and Energy, Department of Environmental and Resource Engineering, Technical University of Denmark, 2800 Lyngby, Denmark; § Climate & Atmosphere Research Centre, The Cyprus Institute, 1645 Nicosia, Cyprus

**Keywords:** VOCs, human emissions, shower, skin, ozone, ozonolysis, lotion

## Abstract

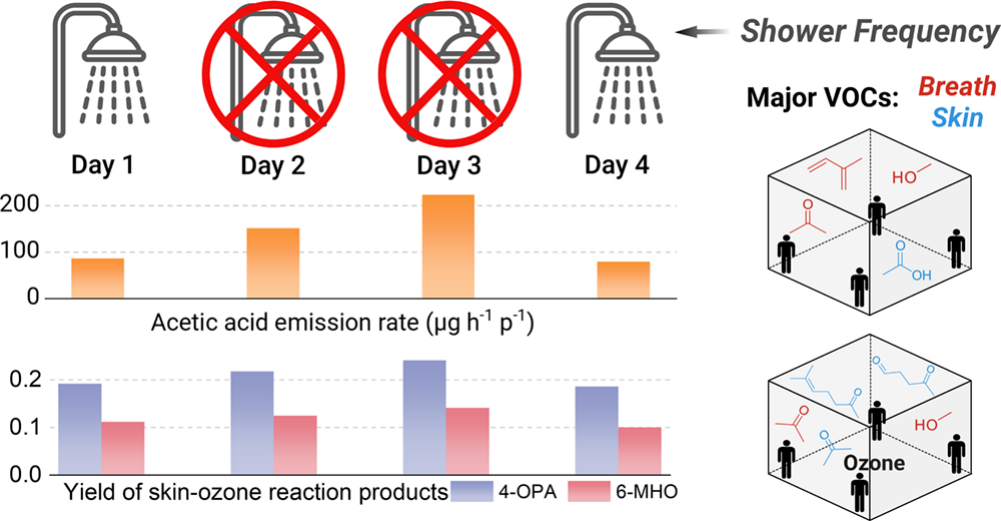

Humans are known to be a continuous and potent indoor source of volatile organic compounds (VOCs). However, little is known about how personal hygiene, in terms of showering frequency, can influence these emissions and their impact on indoor air chemistry involving ozone. In this study, we characterized the VOC composition of the air in a controlled climate chamber (22.5 m^3^ with an air change rate at 3.2 h^–1^) occupied by four male volunteers on successive days under ozone-free (∼0 ppb) and ozone-present (37–40 ppb) conditions. The volunteers either showered the evening prior to the experiments or skipped showering for 24 and 48 h. Reduced shower frequency increased human emissions of gas-phase carboxylic acids, possibly originating from skin bacteria. With ozone present, increasing the number of no-shower days enhanced ozone-skin surface reactions, yielding higher levels of oxidation products. Wearing the same clothing over several days reduced the level of compounds generated from clothing-ozone reactions. When skin lotion was applied, the yield of the skin ozonolysis products decreased, while other compounds increased due to ozone reactions with lotion ingredients. These findings help determine the degree to which personal hygiene choices affect the indoor air composition and indoor air exposures.

## Abstract 2

Abstaining from showering enhances human chemical emissions indoors primarily through carboxylic acids and higher ozone-skin reaction yields of carbonyls.

## 1 Introduction

In recent years, the contribution from human occupants to indoor air volatile organic compounds (VOCs) has drawn increasing attention. Humans innately release thousands of VOCs into the air via multiple pathways, mainly via breath and skin.^1^ In addition, human activities (e.g., cooking and cleaning) expand the variety of VOC emissions and add to indoor concentrations.^2^ One challenge when measuring indoor VOCs is the presence of indoor oxidants (e.g., ozone, hydroxyl radicals, and nitrate radicals), which can react with gas and surface-bound species to generate various primary and secondary VOC oxidation products.^3^ Ozone is considered to be the main indoor oxidant, with sources from both indoors and outdoors.^4^ It can quickly react with compounds containing a carbon–carbon double bond (C=C), generating various smaller, oxygen-containing gas-phase VOCs.^5^ In an occupied indoor environment, the people themselves are likely to be the major ozone removal pathway through reactions with human body surfaces including skin, hair, and clothing, all of which contain skin oil.^6^ Among the skin oil constituents, a unique human sebum component—squalene (C30H50)—is responsible for almost half of the double bonds in skin oil, with further contributions from various fatty acids.^6^ Multiple studies have examined ozone reaction products in laboratory experiments directly from skin oil or skin oil components,^7−13^ from worn clothing,^14−16^ as well as in field studies in occupied aircraft cabins,^17−19^ classrooms,^20−22^ residential homes,^23,24^ offices,^10,25^ and other indoor environments.^26−28^ Furthermore, off-body skin oil present in shed skin flakes or deposited through contact on various indoor surfaces can contribute to ozone removal and product formation even when people are absent.^24,29^ Importantly, some of those ozone-skin oil VOC reaction products have potential adverse health effects.^30^ Therefore, it is important to understand how different parameters, including lifestyle choices, can impact the ozone-skin reactions, the products, and their production rates.

Assuming a constant air change rate (ACR), the abundance of VOCs generated from skin ozonolysis reactions will depend on the indoor ozone level and the amount of available reaction precursors. The skin lipid mixture is continuously generated via sebaceous glands at a rate between 0.4 and 2.5 μg cm^–2^ min^–1^, depending on gender, age, body parts, and so forth.^31^ Accumulated skin lipids can lead to oily skin, which can block pores, encourage microbial growth, and cause potential skin problems and feeling of dissatisfaction.^32,33^ Taking a shower or bathing including washing of the scalp and hair is one of the most common and effective ways to maintain personal hygiene, which helps reduce oiliness of the skin and remove dead skin cells, sweat residues, and adhered pollutants. Therefore, reducing the frequency of taking showers would be expected to alter the overall condition of the skin (skin oil and microbial environment), which, in turn, can affect human VOC emissions and impact the indoor air quality and chemistry, especially when ozone is present. However, no such experiment involving human subjects has been performed to assess this effect in detail.

The Indoor Chemical Human Emissions and Reactivity (ICHEAR 1) project was designed to study the impacts of indoor temperature, relative humidity (RH), clothing coverage, and age on human VOC emissions under ozone-free and ozone-present conditions in a controlled climate chamber.^34,35^ These results demonstrated the impact of occupants on indoor air in isolation from other sources. Therefore, in this follow-up study (ICHEAR 2), the same chamber and experimental procedure were used to investigate the impact of reducing the shower frequency on human VOC emissions. The effect of hygiene on the indoor air VOC composition was investigated under both ozone-free and ozone-present conditions. In addition, the impact of skin lotion application, a common skin care practice, was also studied.

## 2 Materials and Methods

### 2.1 Chamber Setup and Experimental Procedure

The experiments were performed in a 22.5 m^3^ climate chamber located at the Technical University of Denmark. Detailed experimental conditions including temperature, RH, and ozone levels are listed in Table S1. In brief, on Day 1 (benchmark), four volunteers (all male, showered the evening before) wearing identical newly laundered clothing including Bermuda pants, long sleeve shirts, and calf socks entered the chamber in the morning and stayed for 3 h. A short lunch break (∼15 min) was arranged before the volunteers re-entered the chamber in the afternoon for another 2.5 h stay. Around 10 min after re-entering, ozone (∼100 ppb in the supply air) was injected into the chamber through the supply air, causing the mixing ratio of ozone to reach 35–40 ppb at steady state inside the occupied chamber. Prior to the following two consecutive days, Day 2 and Day 3, the volunteers were asked to refrain from showering and wear the same clothing set worn on Day 1. The clothing from Day 1 to Day 3 was only worn during the experiments and was kept in individual sealed bags between experiments. On Day 4, a replicate of the Day 1 experiment (benchmark) was performed (the volunteers showered the evening before and wore a new set of identical clothes). As an extension of this study, the effect of skin lotion application was tested. On Day 7, before entering the chamber in the morning and in the afternoon, the same group of volunteers wiped the exposed skin areas (face, neck, hands, forearm, and lower legs) with a warm wet towel followed by a dry towel and then applied fragrance-free skin lotion on unclothed parts of the body, aiming for an applied amount of 2 mg cm^–2^ (see Table S2 for detailed information). During all experimental sessions, 1.5 h after entering the chamber, the volunteers were asked to stand up and stretch for 10 min; they were otherwise sedentary. The age of the volunteers ranges from 19 to 25 (average of 22.8) with body mass index (BMI) ranging from 21.5 to 26.1 (average of 24.2). Over the entire experimental period, the volunteers were asked to only use the provided fragrance-free soap, fragrance-free shampoo, and toothpaste and avoid drinking alcohol and eating strongly flavored food, as detailed in Bekö et al.^35^ In this study, the last 15 min period before exiting the chamber was considered a steady state. However, due to the different chemical and physical properties of the VOCs analyzed, some species have not reached a steady state in the last 15 min (e.g., secondary ozonolysis products such as 4-oxopentanal (4-OPA)). In order to better quantify the emission rates (ERs) and ozone product yields in this study, any species that did not reach the steady state in the last 15 min were fitted using sigmoidal Gompertz function to derive the steady state values (a detailed description of the application of this fit is provided in the Supporting Information, Table S3, and Figure S1).

In order to better understand the lotion’s role in the indoor air chemistry, a lotion-only experiment was performed. In this experiment, 23.7 g of the lotion was spread on 1.7 m^2^ of the table surface. VOCs inside the chamber were first monitored under no-ozone condition and then under ozone-present condition (supply air ozone mixing ratio ∼100 ppb).

### 2.2 VOC Monitoring

The instrument setup has been described elsewhere.^34^ It included a proton transfer reaction time-of-flight mass spectrometer (PTR-ToF-MS, 8000, IONICON Analytik) and a custom-made fast gas chromatograph–mass spectrometer (fast-GCMS). The chamber exhaust air was sampled through a main high-flow inlet tubing line (fluorinated ethylene propylene, FEP, OD = 1/2 in.) at a flow rate of 13 L min^–1^. The PTR-ToF-MS sampled a substreamflow at ∼20 mL min^–1^ from the main inlet via 0.3 m tubing (OD = 1/8 in.), with a total inlet length of 5.05 m. Similarly, the fast-GC sampled a substream of air through 2 m of tubing (OD = 1/4 in.) with a flow rate of ∼200 mL min^–1^ from the main inlet, with a total inlet length of 6.65 m. The supply air was also periodically sampled through a 1.6 m tubing (OD = 1/2 in.). A three-way valve (Galtek Solenoid Valves, Entegris, Inc.) was used to switch between the chamber air and the supply air.

For PTR-ToF-MS, protonated water (H_3_O^+^) was used as the primary ions. Any VOC with its proton affinity higher than that of water (697 kJ mol^–1^) can be detected at its protonated mass-to-charge ratio (*m*/*z*) due to proton transfer reactions.^36^ In this study, the instrument was operated with a drift tube pressure of 2.2 mbar and a temperature of 60 °C. We measured *m*/*z* up to 500 amu with 20 s time resolution, and the mass resolution was ∼4000 at mass 96 amu. To better quantify the mixing ratios of measured species, 6-methyl-5-hepten-2-one (6-MHO), 4-oxopentanal (4-OPA), and 17 other VOCs were calibrated using pressurized gas cylinders. An experimental transmission curve was derived based on the calibration results and used to quantify further measured species, albeit at somewhat higher uncertainty. Details of the quantification procedure and detection limits are given in the Supporting Information. Chemical formulas were assigned to measured *m*/*z* as previously described.^34^

The primary role of the fast-GCMS was to accurately characterize isoprene, an important reactive VOC emitted from human breath.^1^ Earlier intercomparison experiments have shown that the PTR-ToF-MS measurement of isoprene suffers significant interference from fragments of larger aldehydes.^37^ In addition, the fast-GCMS assisted in the quantification of ketones and aldehydes that share the same exact mass in the PTR-ToF-MS measurement (C_3_H_6_O, C_4_H_6_O, and C_4_H_8_O in this study). In this study, the fast-GCMS collected samples every 3 min using a cryogenic three-step preconcentration followed by GC column separation (DB-624; Agilent Technologies) and detected by a quadrupole mass spectrometer under the selected ion monitoring mode. The operation details and working principles are given elsewhere.^37−39^ The detection limit (LOD) and the total uncertainty for the measured compounds are listed in the Supporting Information (see Table S4).

### 2.3 Other Measurements

A Jelight 600 UV ozone generator (Jelight Co., Inc.) was used to generate ozone in the supply air. A 2B Technologies model 205 ozone monitor (2B Technologies) measured the ozone level alternately in the supply air and in the chamber. Further details on those instruments are given in Bekö et al.^35^

### 2.4 Ozone Product Yield Calculation

To better understand how personal hygiene can influence the products from ozone reactions, we calculated the overall yields of those products (*Y*_*i* (overall)_) by using eq 1.
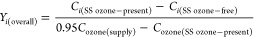
1*C*_*i*(SS ozone–present)_ and *C*_*i*(SS ozone–free)_ represent the mixing ratios of an ozone product *i* at steady state (SS) under the ozone-present condition and the ozone-free condition, respectively. *C*_ozone(supply)_ refers to the ozone mixing ratio measured in the supply air, and 0.95 was used to correct the ozone loss to the surfaces of the unoccupied chamber as determined earlier (5%).^35^*C*_ozone(SS ozone–present)_ is the steady-state ozone mixing ratio under the ozone-present condition. It is worth noticing that the yield derived from eq 1 should be considered the upper limit due to potential low levels of VOCs generated from the chamber surface reacting with ozone (<5%).

For the main squalene ozonolysis products 6-MHO and 4-OPA, we used a mass balance model modified from Salvador et al.^40^ to derive the surface yield and gas-phase contribution from their precursors. During the steady-state condition, we assume that the production of product *i* (6-MHO or 4-OPA) is dominated by the ozone-surface reaction and the ozone-gas-phase-precursor reactions (denoted *j*), which is balanced by the loss of product *i* through ventilation and reaction with ozone. This can be described in eq 2.
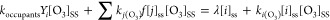
2*Y*_*i*_ is the surface yield of product *i. k*_occupants_ is the first-order ozone removal rate coefficient by human occupants (mainly to skin and clothing). The calculation details of this parameter can be found in the Supporting Information. [O_3_]_SS_, [*j*]_ss_, and [*i*]_ss_ refer to steady-state concentrations of the O_3_, precursor *j*, and product *i*, respectively. *k*_*j*(O_3_)_ is the rate coefficient of precursor *j* reacting with O_3_, and *f* refers to the branching ratio of product *i* generated from precursor *j* reacting with O_3_. λ represents the ACR (3.2 h^–1^). *k*_*i*(O_3_)_ is the rate coefficient of product *i* reacting with O_3_. Detailed equations and coefficients used for 6-MHO and 4-OPA surface yield calculation can be found in the Supporting Information (mass balance model and Table S5).

### 2.5 Statistics

A Pearson correlation analysis was applied to identify correlations between VOCs measured in the experiments. A *p*-value <0.05 indicates that the correlation is significant. One-way analysis of variance (ANOVA) was applied to identify the statistical differences between benchmark experiments and no-shower experiments, where a posthoc test (Tukey test) was followed for the mean comparisons. A *p*-value <0.05 represents a significant difference. The analysis was performed with OriginLab (version: 2021b).

## 3 Results and Discussion

### 3.1 Benchmark Emission Rates Compared to the Previous ICHEAR Study

The ERs of VOCs were calculated analogously to our previously published study^34^ to allow comparison. In order to be consistent in terms of conditions, here, we compare the average of the benchmark and its replicate conditions (Experiment No. 1 on Day 1 and 5 on Day 4 in Table S1) to the whole-body ERs derived from benchmark groups in our previous study.

The VOC composition of the whole-body ERs from this study agrees well with our previous study (see Figure S2 in the Supporting Information).^34^ Under the ozone-free condition, acetone, methanol, and isoprene had the highest ERs accounting for 32, 18, and 6% of the measured compounds, respectively. Among the top 10 contributors to the total emission, seven species were identical to those in the previous study. Meanwhile, the total ER (3350 μg h^–1^ p^–1^) from this study was higher than the value we obtained before (2180 ± 620 μg h^–1^ p^–1^).^34^ The increase was mainly introduced by methanol and other species belonging to the chemical formula groups C_*x*_H_*y*_, C_*x*_H_*y*_O, and C_*x*_H_*y*_O_2_, as shown in Figure S2c. Gender difference may play a role in this study, as four male volunteers participated in the experiments instead of two male and two female volunteers as in the previous study. Shetewi et al. observed higher levels of aldehydes, ketones, and acids in male participant forearm samples than in female participants.^41^ However, the deviation could also be due to interindividual differences, especially for methanol in breath.^42^

When ozone was present in the chamber, the total ER increased ∼2.5 times (8708 μg h^–1^ p^–1^) compared to the total ER under the ozone-free condition. This value is almost double that of the ozone-present total ER (4600 ± 501 μg h^–1^ p^–1^) obtained in our previous work.^34^ 4-OPA, methanol, and other species in the group of C_*x*_H_*y*_O, C_*x*_H*y*, and C_*x*_H_*y*_O_2_ contributed the most to the increase (see Figure S2b,d). Compared to women, men generally have higher sebum production rates,^32,43^ leading to more abundant precursors (e.g., squalene and fatty acids) of skin-ozone reaction products. Therefore, the higher ERs of 4-OPA and other ozonolysis products in this study are likely due to gender-related skin oil abundance and composition differences.

### 3.2 The Effect of Hygiene Status on VOC Emissions

As taking a shower mainly impacts the skin surface condition, VOCs associated with dermal emissions were expected to vary more according to the state of hygiene than breath-borne compounds. Therefore, the major breath-borne VOCs (methanol, acetone, isoprene, and dimethyl sulfide)^34^ and two skin-associated VOCs (acetic acid and 4-OPA)^34,44^ were selected to verify this assumption. As expected, VOCs originating primarily from human breath did not show a clear association with hygiene status (see Figure S3). Isoprene and dimethyl sulfide were at similar levels throughout the four experiments. For methanol, the volunteers emitted most on experiment Day 2 (1 day without shower) followed by Day 1 (benchmark), Day 3 (2 days without shower), and Day 4 (benchmark, replicate), which might be due to food/drink consumption containing methanol (e.g., juices) prior to the experiments at breakfast, or ingredients ingested at breakfast that can be subsequently metabolized to methanol.^42^ For acetone, the highest concentration was observed on Day 1 followed by Day 2, Day 3, and Day 4. Acetone concentrations in human breath can vary substantially even between samples taken from the same individual,^45^ and they vary with changing body energy balance.^46^ Although acetone is one of the squalene ozonolysis compounds,^10^ the mixing ratios during the ozone-present condition were not consistently higher during no-shower days than during benchmark days (Figure S3), likely because they were driven by breath emissions.

**Figure 1:**
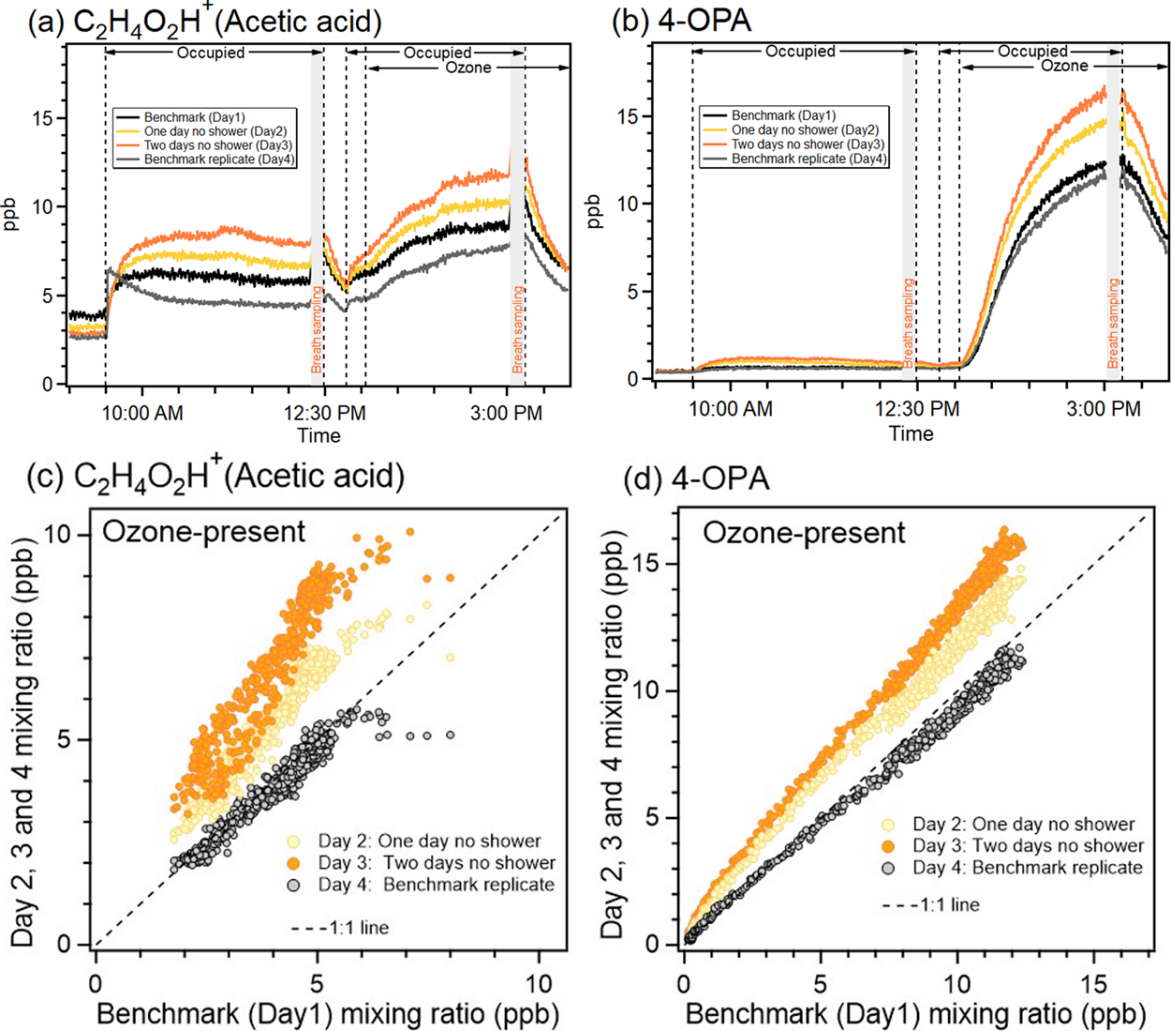
Time series of mixing ratios for (a) C_2_H_4_O_2_H^+^ (acetic acid) and (b) 4-OPA during the hygiene experiments, and scatter plots of benchmark (Day 1) versus other experiments (benchmark replicate and no-shower experiments) for (c) C_2_H_4_O_2_H^+^ (acetic acid) and (d) 4-OPA. The shaded gaps represent the time period when the PTR-MS measured breath.

In contrast, one of the major VOCs from skin and ozone-skin reactions, acetic acid, clearly shows higher steady-state mixing ratios on no-shower days (Figure 1a). Similarly, the skin ozonolysis product 4-OPA increased with longer time since the last shower (Figure 1b). In order to better evaluate the reproducibility of the experiments, for acetic acid and 4-OPA, we correlate their mixing ratios (background level subtracted) from the benchmark experiment (Day 1) with those from three other experiments under ozone-present conditions (Figure 1c,d). The benchmark replicate showed good agreement with the benchmark for acetic acid (slope: 0.90, *R*^2^: 0.92) and 4-OPA (slope: 0.92, *R*^2^: 0.99). Acetic acid and 4-OPA in two other experiments (1 day and 2 days without shower) also correlated well with their mixing ratios in the benchmark experiment. Therefore, the results indicate that the experiment in our study is reproducible, especially when ozone is present in the chamber. Meanwhile, the scatter deviation observed from the no-shower experiments can be clearly separated from the benchmark replicate experiment, as shown in Figure 1c,d, indicating that the hygiene intervention may have played a role.

In order to test the effect of skin hygiene on human VOC emissions while considering potential intragroup variability across experimental days (reproducibility), we performed a variability test by calculating the percentage change between the benchmark experiment (Day 1) and its replicate (Day 4) from this study, the percentage change between four benchmark/replicate experiment pairs in the previous ICHEAR project performed using the same experimental procedure,^34^ and the percentage change between the hygiene intervention (no shower) days and the benchmark conditions in this study (Day 1 and Day 4 experiment, respectively). This allows us to statistically compare if there is any difference in terms of the relative change between each two conditions (benchmark, one-day no shower and two-days no shower). Details of this approach are described in the Supporting Information, and the results are shown in Table S6. In what follows, the term “overall benchmark” will be used to represent the benchmark experiments and their replicates, both from this study and from the previous ICHEAR study. In summary, combining the experiments in this study with previous benchmark experiments with different individuals, we can gauge the robustness of the results.

#### 3.2.1 Ozone-Free Condition

For the hygiene experiments, after subtracting the top three contributors (breath-borne: acetone, methanol, and isoprene), the highest total ER (2079 μg h^–1^ p^–1^) was observed on Day 3 (2 days without shower), which is 43% higher than the total ER of the benchmarks in this study. This relative increase is significantly higher than the mean relative change derived from the overall benchmarks (−0.7%, *p* < 0.01, Table S6). Besides those top three compounds, acetic acid was the most emitted compound. Compared to the benchmarks (Day 1 and Day 4 experiments), it increased by 84% after one day and 172% after two days without showering, which were significantly higher than the relative change across overall benchmarks (*p* < 0.001). Additionally, there is also significant difference of the relative change between one-day no-shower condition and two-day no-shower condition for acetic acid (*p* < 0.001). We further classified emitted species in chemical family groups, as in our previous study,^34^ as shown in Figure S4a in the Supporting Information and correlated the total ER of each group with the ER of acetic acid. Only the C_*x*_H_*y*_O_2_ (species containing two oxygens) group showed significant correlations with acetic acid (*r*^2^ > 0.90, *p* < 0.05). The corresponding relative change in the ER for one-day no shower vs benchmarks, two-days no shower vs benchmarks, and one-day no shower vs two-days no shower significantly differed between two of these pairs of conditions (Table S6). In the group of C_*x*_H_*y*_O_2_, a series of compounds with the chemical formula C_*n*_H_2*n*_O_2_ (*n* = 2, 3, 4, 5, 6, 8 and 9) were ranked among the top 10 compounds with the highest emissions. Acetic acid together with other carboxylic acids have been previously measured in human skin volatiles and are known to be degradation products of long-chain acids catabolized from the skin lipid by bacteria in human sebum.^47^ Misztal et al. measured VOCs emitted from skin-associated microbial species using the same type of the instrument as we used in our study and found several ions with the formula C_*n*_H_2*n*_O_2_H^+^ among the most abundantly emitted ions.^48^ Therefore, the species falling within the C_*n*_H_2*n*_O_2_ chemical group are likely to be carboxylic acids. Abstaining from showering possibly allows bacteria to accumulate and actively generate volatile carboxylic acids on the skin surface. Interestingly, some of these carboxylic acids are known to contribute to the human body odor and potentially attract biting insects such as mosquitos.^47^

#### 3.2.2 Ozone-Present Condition

For the ozone-present condition, the difference in the total delta ER (the difference between the ER during steady state with ozone present and absent) rose by 22% after 2 days without shower compared to benchmarks in this study, which is significantly higher than the mean relative change derived from the overall benchmarks (Table S6). The increase was generated mainly by 4-OPA, 6-MHO, and other carbonyl compounds known to be products of skin-ozone reactions^10^ (Figure S4b). To better understand the effect of hygiene status on ozone-skin reactions, the overall yields *Y*_*i*(overall)_ of different species were calculated based on eq 1. The yields of common reaction products among the top 20 with the highest yields are listed in Table 1. The overall yields of 4-OPA, 6-MHO, and 1,4-butanedial increased with more time since last shower. All three compounds showed significant relative increase of the yield after 2 days without shower compared to the relative change across overall benchmarks (*p* < 0.05). For 6-MHO and 4-OPA, the relative increase of the overall yield after one-day without shower also differed significantly from the relative change across overall benchmarks (*p* < 0.05).

**Table 1:** Overall Yields of Common Ozone Reaction Products (Each within the Top 20 Species by Yield) during the Hygiene Experiments (*N* = 4) with Four Male Volunteers in a 22.5 m^3^ Chamber with an Air Change Rate of 3.2 h^–1^^a^

*m*/*z* (H^+^)	compound assignment	benchmark (day 1)	one-day no shower (day 2)	two-days no shower (day 3)	benchmark replicate (day 4)
59.049	acetone^b^	0.209	0.311	0.293	0.306
101.060	4-OPA^b^	0.192	**0.217**^c^	**0.241**^c^	0.186
127.112	6-MHO^b^	0.112	**0.125**	**0.141**	0.100
61.028	acetic acid	0.053	0.059	0.068	0.060
157.159	decanal	0.049	0.046	0.050	0.043
143.107	OH-6MHO	0.021	0.026	0.029	0.028
87.044	1,4-butanedial	0.024	0.027	**0.030**	0.025
45.033	acetaldehyde^b^	0.026	0.030	0.029	0.028
75.044	hydroxy acetone/propionic acid	0.018	0.019	0.022	0.020
143.143	nonanal	0.033	**0.023**	**0.019**	0.032

a Values in bold indicate that the mean relative change of no-shower experiments compared to the benchmarks is significantly different from the mean relative change across overall benchmarks (|*p* < 0.05). Several fragments were also ranked within the top 20 species with the highest yields but are not shown here.

b The mixing ratios of this compound were calibrated using gas standard.

c There is significant difference between the relative change of one-day no-shower experiment and the relative change of two-days no-shower experiment.

**Figure 2:**
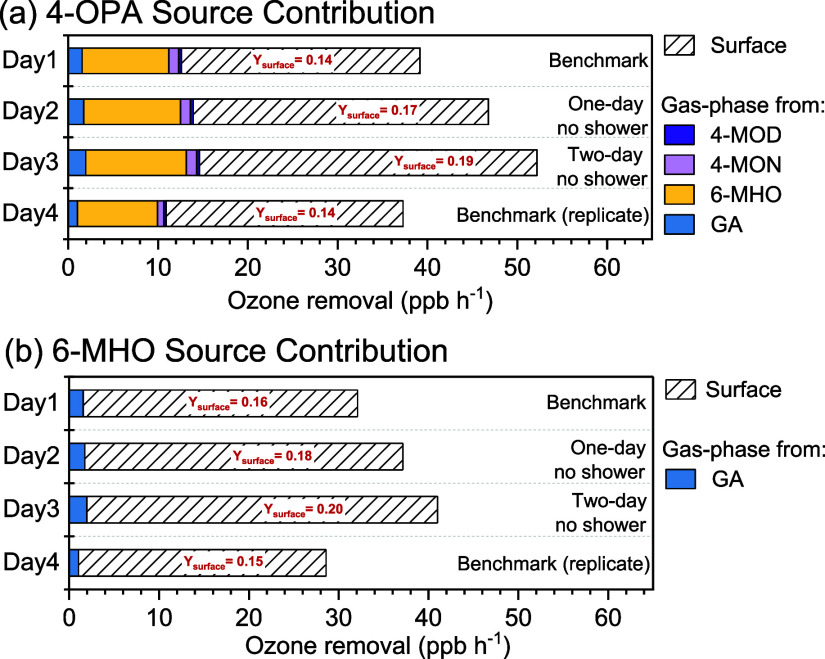
Source contribution from the surface and gas phases to (a) 4-OPA and (b) 6-MHO during the hygiene experiments.

Since the major squalene ozonolysis products 4-OPA and 6-MHO were well-quantified and their reaction mechanisms are well-understood, we applied a mass balance model to further elucidate their source contribution with changing hygiene status. More importantly, surface yields are less sensitive to the environmental parameters,^49^ which allows comparison with other studies. The results showed that, on average, ozone was removed mostly by the human body surfaces (∼60%) followed by the ventilation (∼36%), while removal by reactions with gas-phase VOCs only accounted for 4% (see Table S7 in the Supporting Information). As shown in Figure 2, for 4-OPA, 67–71% of the concentration was attributed to surface reaction, while the gas-phase secondary formation accounted for 29–33%, mostly from 6-MHO. For 6-MHO, almost 95% was attributed to surface reaction with only ∼5% from the gas-phase reaction of ozone with geranyl acetone (GA). The contribution of surface reactions to both compounds increased with longer time since showering. The surface yield of 4-OPA increased from 0.14 on benchmark days to 0.17 when showering was skipped for 1 day and further increased to 0.19 when showering stopped for 2 days. The relative increase of the 4-OPA surface yield due to skipping shower significantly differed from the relative change across overall benchmarks (*p* < 0.001, Table S6). Meanwhile, the relative increase of the surface yield also significantly differed between one-day no-shower and two-day no-shower conditions. Similar values were found for 6-MHO (see Figure 2b), while only the surface yield from two-day no-shower condition showed significant relative increase compared to the overall benchmarks (*p* < 0.005, Table S6). The surface yield of 6-MHO derived in our study is in agreement with Morrison et al. (0.219 ± 0.081)^50^ but higher than the value obtained from Qu et al. (0.094 ± 0.013),^51^ while the surface yield of 4-OPA calculated in this study is generally much higher than those two studies (0.007 ± 0.019,^50^ 0.046 to 0.054, which were calculated using the ozone dependency reported by Qu et al.^51^). As discussed in both studies, values can differ from study to study due to different experimental conditions including calculation methods, chamber types, sample types, individual variability, as well as different quantification techniques.^50,51^

The above results indicate that hygiene status (time since the shower) can impact ozone-skin surface reactions. As the overall yield and surface yield of major squalene ozonolysis gas-phase products (6-MHO and 4-OPA) increase with a longer time since showering, the accumulation of their precursor, squalene, on skin would be expected. However, studies have shown that there is an equilibrium value for the amount of sebum spread over the skin without any removal process, which differs among different body parts and varies from person to person, mainly depending on the number of sebaceous glands.^32,52^ After removal (cleaning), the equilibrium value can be quickly recovered within 2 h in all seborrheic areas (face and upper back).^53^ Therefore, in our study, squalene, the main compound in human sebum,^54^ should be present at a similar level during one-day and two-day no-shower experiments which were performed more than 24 and 48 h after taking shower. Squalene ozonolysis leads to a complex set of reactions, especially in the condensed phase, generating products mostly containing peroxy, hydroxyl, and ether functional groups.^55^ As the volunteers were continuously exposed to air that most likely contained low levels of ozone prior to the experiments, primary and secondary squalene ozonolysis products present in the condensed phase may have accumulated with a longer time since showering, resulting in more precursors for further gas-phase oxidation products measured in the chamber.

In particular, as human face and scalp are the most sebum-containing parts of the skin^56^ and the volunteers may wash their face between experiments, human scalp and hair may have contributed substantially to the squalene ozonolysis products in our study. Pandrangi et al. measured higher ozone uptake and sebum-ozonolysis products from unwashed hair compared to washed hair exposed to ozone.^57^ Punyani et al. observed significantly higher amount of oxidized lipids in samples from both scalp and hair close to scalp after refraining from shower for 7 days compared to daily washing, indicating the influence of low hair wash frequency on the chemical composition of the sebum.^33^ Detailed chemical analysis of skin and hair samples from different body parts before and after ozone exposure following different lengths of time since showering could help better understand sebum oxidation in relation to showering frequency.

Although acetone, hydroxyacetone, and 1-hydroxy-6-methyl-5-hepten-2-one (OH-6MHO) are also squalene ozonolysis products,^10^ no consistently increasing trend with no-shower days was observed. As the acetone level in breath might have changed in the afternoon when ozone was present due to lunchtime food intake, the acetone increase from ozonolysis is more uncertain, a limitation that was also observed in our previous study.^34^

For decanal, the yield of four males ranged from 0.043 to 0.050 and showed no clear difference between no-shower days and benchmark days (Table 1). Decanal is known to be generated from unsaturated fatty acids (UFAs) contained in skin lipids.^6,10^ UFAs are mainly present in human epidermal lipid (the outermost layer exposed to the air),^54^ which has much lower mass compared to the sebum where squalene is mainly located (∼30 times less on the forehead).^58^ This difference in mass could be the reason for not seeing a clear impact of longer time without shower on decanal yields. Therefore, no clear dependency of decanal on hygiene status (up to 2 days without shower) was observed.

Interestingly, the overall yield of nonanal showed a significant decrease with increasing time since shower (Table 1). The relative changes in the yields for one-day and two-day no-shower experiments (relative to the benchmarks) were significantly higher compared to the relative change in the yields across overall benchmarks (Table S6). Nonanal has been found to be generated from ozone reacting with oleic acid,^9^ which only accounts for a minor fraction of human skin lipids.^54^ Previous studies have also indicated that nonanal can result from skin-ozone reactions involving accumulated cooking oil on human skin.^24,50^ Therefore, increasing nonanal should have been observed with a longer time without shower, but the opposite trend occurred. Nonanal has been also measured when cotton material was exposed to ozone.^14,15^ In our previous study, when the volunteers took out clean cotton shirts from sealed bags in the chamber to wear under the ozone-present condition, nonanal levels increased, while squalene ozonolysis products decreased. This strongly suggested the presence of nonanal precursors in the cotton fabric.^34^ In the current study, the clothing sets worn by the volunteers on benchmark Day 1 were also worn during Day 2 and Day 3. Therefore, in our study, the decrease in nonanal yields is more likely due to the precursors of nonanal present in the clothing being depleted by ozone exposure over the course of the experiments.

### 3.3 The Effect of Skin Lotion on VOC Emissions

Under the ozone-free condition, the lotion-on-skin experiment had the highest total ER of 10115 μg h^–1^ p^–1^ among all experiments. The top three contributors, C_8_H_8_O, C_8_H_10_O and C_6_H_6_O, accounted in total for 66% of the total ER (see Figure S5 in the Supporting Information). These species showed a sharp increase after the volunteers entered the chamber both in the morning and in the afternoon, and then started to decrease after about 30 min (see Figure S6 in the Supporting Information). These species were, therefore, deemed to be direct emissions from the applied lotion, which was confirmed by the lotion-only experiment (see Figure S7 in the Supporting Information). One component named phenoxyethanol (C_8_H_10_O_2_) on the ingredients list is likely to be the major contributor to those top three species with C_8_H_8_O and C_6_H_6_O likely being its fragments in the PTR-MS. The mixing ratios of those three masses were almost 2 orders of magnitude lower during benchmark conditions compared to the lotion-on-skin experiment but showed a slight increase after ozone was injected, as shown in Figure S6. Other ozone oxidation products with the same chemical formula (isomeric compounds) landing on those three masses can be the reason. After the top three emitted species that are presumably components in the lotion were excluded, the total ER of the lotion-on-skin experiment was comparable to the benchmark ER, and the top contributors were breath-born compounds. Acetic acid had similar levels as under benchmark conditions; no clear effect of lotion on dermal emission could be identified when ozone was absent.

**Figure 3:**
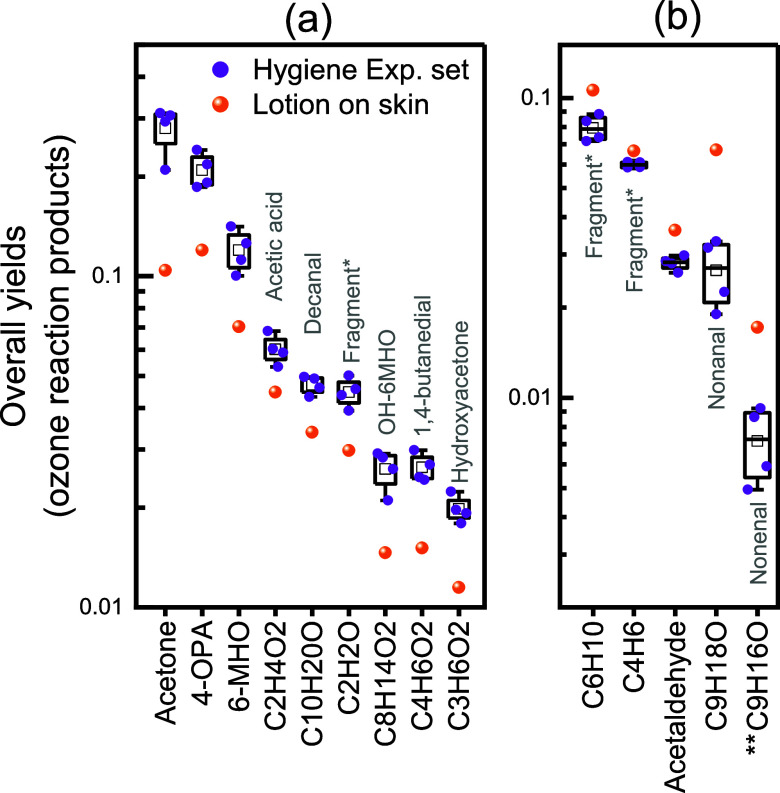
Overall yields of common species among the top 20 with the highest yield during the four hygiene experiments (boxes and purple dots) and their corresponding yields during the lotion-on-skin experiment (orange circles): species showing lower yields (a) and higher yields (b) when lotion was applied on the skin compared to the no-lotion condition (four hygiene experiments). The box plots stand for data in the range of 25 to 75%. The square in the box represents the mean values. The whiskers represent the minimum and the maximum. * indicates that the species is more likely to be a fragment ion from bigger compounds. **C_9_H_16_O (nonenal) was not a common species ranked among the top 20 with the highest yield across all experiments.

With ozone present in the chamber, the aforementioned three lotion-related species still dominated the total ER due to the lunchtime reapplication of the lotion. By excluding lotion-emitted species, the total ER was ∼8150 μg h^–1^ p^–1^, which was slightly lower than the total ER of the benchmark experiments. The rest of the top 10 emitted species were similar to the benchmarks (see Figures S2b and S5b in the Supporting Information). To better evaluate the effect of the skin lotion on VOC emissions, we calculated the overall yields of measured VOCs for the lotion-on-skin experiment and compared them to the overall yields obtained from the four hygiene experiments. Among the top 20 species by yield, there are in total 13 species in common for all experiments. Those 13 species were further divided into two groups depending on whether the overall yield in the lotion-on-skin experiment is higher or lower than the corresponding overall yield in the hygiene experiments, as shown in Figure 3a,b. Species that showed lower yields during the lotion-on-skin experiment compared to the hygiene experiments (see Figure 3a) are mainly compounds generated from skin lipids reacting with ozone, as discussed in Section 3.2.2. This indicates that lotion coverage lowered the generation of skin-ozone reaction products. Our results agree with a recent study where reduced yields of skin lipid ozonolysis products were observed by applying different types of lotion.^59^ It is worth noting that the ozone removal rates were similar between experiments with and without lotion application. This is mainly because the ozone removal to the surface is driven by the resistance to mass transport across the boundary layer of air adjacent to the surface.^60^ Therefore, it is likely that the applied lotion competes with skin lipids to react with ozone. Nonanal and aldehyde-related fragments showed higher overall yields when lotion was applied on the skin (Figure 3b). Based on the time series of those species (Figure S8 in Supporting Information), they had a comparable level to the benchmark conditions when ozone was absent in the chamber but increased to higher levels when ozone was present. The lotion used in this study has natural ingredients of rapeseed oil and shea butter (within the top five ingredients), both of which contain large amounts of unsaturated fatty acids, mainly oleic acid and linoleic acid.^61,62^ When reacting with ozone, the main product of oleic acid is nonanal, and the main products of linoleic acid are nonenal and hexanal.^9^ During the lotion-only experiment, both nonanal and nonenal showed a slight increase when ozone was introduced (Figure S7). Therefore, part of the ozone was consumed by the lotion ingredients, leading to higher nonanal, nonenal, and aldehyde fragments (C_6_H_10_ and C_4_H_6_ are the major fragments in PTR-MS for nonanal and hexanal, respectively^63^). Acetaldehyde is a product of skin lipid reacting with ozone and was also found to be present as a primary compound emitted from the lotion (Figure S7). For the lotion-on-skin experiment, acetaldehyde reached a higher steady-state mixing ratio in the ozone-present condition compared to the no-ozone condition (Figure S8). The yield of acetaldehyde in the lotion-on-skin experiment was, thus, influenced by both direct emissions from the lotion and ozone reactions on the skin, with the former contributing more to the overall yield.

### 3.4 Implication of the Study

According to a survey taken by over 6000 people from 14 countries, the global average shower frequency is around once per day, ranging from ∼5 times per week in Japan and China to ∼12 times per week in Brazil.^64^ Our study shows that in an ozone-free indoor environment, the ER of the human body (skin + clothing + hair) increased by 20% at a shower frequency of 4 times per week and by 42% at a shower frequency of 3 times per week compared to 7 times per week. When ∼37 ppb ozone was present indoors, the total ER excluding major VOCs from the breath was elevated by an additional 9 and 18% for 4 showers per week and 3 showers per week, respectively. In real-world indoor environments, the indoor ozone levels are lower compared to the ozone levels in our study (on average 4–6 ppb^4^), leading to correspondingly lower ERs of skin ozonolysis products. On the other hand, the increasing trend of those products (e.g., 4-OPA and 6-MHO) with lower shower frequency would still be noticeable, as the surface yields of those products were higher at low shower frequency. In addition, acids and aldehydes usually have very low odor thresholds, for example, 6 ppb for acetic acid^65^ and ∼40 ppt for decanal.^66^ They were measured at such levels in this study, and their levels were higher at lower shower frequency. This indicates that shower frequency is linked to indoor odor contributed by humans, which can be potentially enhanced due to the presence of ozone presence. Therefore, the shower frequency should be considered as one of the factors that can influence the indoor VOC composition and chemistry. Since the study was performed with a limited number of volunteers (constrained by the financing and time of the project), we could not address the potential individual variability in terms of VOC emissions and ozone oxidation product yields. Future experiments with more diverse volunteers (e.g., age, BMI, gender, etc.) are needed to explore further dependencies.

## Supporting Information Available

Further details of quantification of VOCs measured by PTR-ToF-MS; steady-state level determination for species not reaching steady state in the last 15 min; mass balance model; the variability calculation for benchmarks and no-shower experiments; Supporting Tables S1 to S7 and Figures S1 to S8 (PDF)
