# Supplementary material for: How Does Personal Hygiene Influence Indoor Air Quality?
Source: Environ Sci Technol. 2024 May 23;58(22):9750–9. doi: 10.1021/acs.est.4c01698 (PMC11155237; doi:10.1021/acs.est.4c01698)
Supplement: Supplementary file 1 — es4c01698_si_001.pdf [file es4c01698_si_001.pdf]

Supporting Information

How does personal hygiene influence indoor air quality?

Nijing Wang[1], Tatjana Müller[1], Lisa Ernle[1], Gabriel Bekö[2], Pawel Wargocki[2], Jonathan Williams[1, 3]*

[1]Atmospheric Chemistry Department, Max Planck Institute for Chemistry, 55128 Mainz, German

[2]International Centre for Indoor Environment and Energy, Department of Environmental and Resource Engineering, Technical University of Denmark, 2800 Lyngby, Denmark

[3]Climate & Atmosphere Research Centre, The Cyprus Institute, 1645 Nicosia, Cyprus

*corresponding author: Nijing Wang (nijing.wang@mpic.de)

Pages: S1 – S18

Tables: S1, S2, S3, S4, S5, S6, S7

Figures: S1, S2, S3, S4, S5, S6, S7, S8

1. Quantification of VOCs measured by PTR-ToF-MS

A standard gas mixture containing 17 compounds (Apel-Riemer Environmental Inc.) was used for the calibration during the experiments. In total, three calibrations under varying humidity levels were performed. 6-methyl-5-hepten-2-one (6-MHO) and 4-oxopentanal (4-OPA) were calibrated after the campaign in the lab using gas cylinders (Westfalen AG). Table S4 shows the compound list with their limits of detections and total uncertainty. Based on the calibration results, an experimental transmission curve was obtained to quantify other measured masses using the theoretical method[1–3] by using a constant proton transfer reaction rate coefficient of 2.5×10^{-9} cm^3 $molecule^{-1}$ s^{-1}. The uncertainty of these species mainly comes from the difference of the exact rate coefficient, which is within ~ 50%.[2,4]

In order to filter out the VOC species emitted due to human occupancy; a filter was applied based on the mixing ratios. Only VOCs with the difference between steady-state levels in the occupied chamber and the empty chamber larger than two times the standard deviation of the empty chamber level were considered to be associated to human occupancy. In addition, a unified mass list was created based on filtered VOC species from the four hygiene experiments to study the common human-associated VOCs.

2. Steady-state level determination for species not reaching steady state in the last 15 minutes

Due to the different chemical and physical properties of VOCs analyzed, the time needed to reach the steady state condition varies. In particular, when ozone was present in the chamber, some VOC species were newly generated in the chamber, not necessarily reaching steady state before the end of the experiments. For example, it was found that 4-OPA, a secondary squalene ozonolysis product, was often found to be taking longer to reach steady state compared to 6-MHO (primary and secondary product)[5]. In this study, 4-OPA kept increasing until the volunteers exited the chamber (Figure 1b). In order to identify any other species that similarly did not reach steady state in the last 15 minutes before volunteers left the chamber, Pearson correlation analysis was performed between 4-OPA and all other species over the time period when ozone was present in the chamber. For those species significantly correlating with 4-OPA ($R^2 \geqslant 0.90$, $p \leqslant 0.01$), we assume they too may not have reached steady-state in the last 15 minutes. In these cases, we applied sigmoidal fitting to those species to estimate the steady-state values using OriginLab

(OrigninPro 2021b). The Gompertz function was found to be the best fitting for the selected time period, where those 4-OPA correlated species could be all successfully fitted.

Figure S1 shows an example of sigmoidal Gompertz fitting for 4-OPA, where the estimated steady-state mixing ratio was slightly higher than the 15-minute-averaged value (2,4%). For other species that may not have reached steady state, the steady-state mixing ratios derived from the fitting results were mostly around or below 5% higher compared to the last 15-minute-averaged values, as shown in Table S3. Only one species $C_5H_6O_2H^+$ (*m/z* 99.0441) showed much higher steady-state mixing ratio than the last 15-minute-averaged level (14% to 21%). In order to further improve the calculation of the emission rates and the ozone product yields that are based on steady state condition, sigmoidal fitting derived steady-state mixing ratios during the ozone-present condition were used for compounds listed in Table S3. As a result, the total emission rate (ER) only increased by 0.5% ~ 1.5% (83 µg h^{-1} p^{-1} to 141 µg h^{-1} p^{-1}) for experiments included in this study (Table S1), compared to using the last 15-minute average mixing ratios. The increase due to steady-state fitting is much less than the total ER difference between benchmark and its replicate (1331 µg h^{-1} p^{-1} between Exp. 1 on Day 1 and Exp. 5 on Day 4). Therefore, we can conclude that for most of the VOCs measured, the steady-state condition has been reached.

3. Mass balance model

- First-order ozone removal rate coefficient by occupants ($k_{occupants}$)

During steady state, the ozone emission rate is identical to the ozone removal rates from all pathways. In our study, the main ozone removal pathways in the chamber are 1) ventilation; 2) gas-phase reactions; 3) occupants (mainly skin and clothing); 4) chamber surface. Therefore, the ozone emission rate (ER, ppb h^{-1}) can be described as the following Eq. S1.

$$\boldsymbol{ER_{O_3} = \lambda[O_3]_{ss} + k_{occupants}[O_3]_{SS} + \sum_{gas-phase} k_{i(O_3)}[i]_{SS}[O_3]_{SS} + k_{chamber}[O_3]_{SS}} \qquad \text{Eq. S1}$$

ER_{O_3} can be derived from the measured ozone level in the supply air multiplied by the air change rate ($\lambda = 3.2\ h^{-1}$). $[O_3]_{ss}$ is the ozone mixing ratio during the steady state. $[i]_{SS}$ refers to the mean mixing ratio (ppb) of O_3-reactive VOCs over the steady-state period. Here, we included 9 VOCs containing a carbon-carbon double bond (see Table S5), which were the main O_3-reactive compounds[6]. $k_{i(O_3)}$ represents the gas-phase second-order reaction rate coefficient of VOC i reacting with ozone (listed in Table S5). $k_{chamber}$ is the removal rate of chamber surface. As the

experiments were performed in the same chamber that our previous study was performed, the same value of $k_{chamber}$ (0.17 h^{-1}) was used[7]. The first-order ozone removal rate coefficient by occupants ($k_{occupants}$) can be derived from Eq. S1. The mass balance model terms for ozone removal in the hygiene experiments are listed in Table S7.

- 6-MHO and 4-OPA

For 6-MHO, the gas-phase precursor is geranyl acetone (GA)[5]. For 4-OPA, the gas-phase precursors include GA, 6-MHO, 4-methyl-8-oxo-4-nonenal (4-MON) and 4-Methyl-4-octene-1,8-dial (4-MOD) [5]. During the steady-state condition, the mass balance for 6-MHO and 4-OPA can be described by Eq. S2 and S3, respectively.

$$k_{occupants}Y_{6MHO}[O_3]_{SS} + 0.5k_{GA(O_3)}[GA]_{ss}[O_3]_{SS} = \lambda[6MHO]_{ss} + k_{6MHO(O_3)}[6MHO]_{ss}[O_3]_{SS} \quad \text{Eq. S2}$$

$$k_{occupants}Y_{4OPA}[O_3]_{SS} + 0.5k_{GA(O_3)}[GA]_{ss}[O_3]_{SS} + k_{6MHO(O_3)}[6MHO]_{ss}[O_3]_{SS} + 2k_{4MON(O_3)}[4MON]_{ss}[O_3]_{SS} + k_{4MOD(O_3)}[4MOD]_{ss}[O_3]_{SS} = \lambda[4OPA]_{ss} \quad \text{Eq. S3}$$

Y_{6MHO} and Y_{4OPA} are the surface yields of 6-MHO and 4-OPA, respectively. The second-order rate coefficients of VOCs with ozone in Eq. S1, S2 and S3 are listed in Table S5. 0.5 and 2 refer to the branching ratios of 6-MHO or 4-OPA generated from their precursors reacting with O_3. Values were taken from our previous study[6].

4. Variability calculation for benchmarks and no-shower experiments

When comparing the relative change between no-shower experiments and benchmark experiments, the intra-group variability (same volunteers performing replicate experiments) should be considered to identify if the difference is significant. Therefore, we evaluated the variability (reproducibility) using the benchmark and its replicate experiment (Exp. 1 and Exp .5) and four pairs of benchmark-replicate from the previous ICHEAR project involving three different volunteer groups with the same experiment procedure (whole day experiment with ozone present in the afternoon): Exp. 6 vs. Exp. 21, Exp. 8 vs. Exp. 23, Exp. 16 vs. Exp.25 and Exp. 18 vs. Exp.26 [8]. As the absolute change among different experiment pairs may vary due to various factors (gender, clothing and age), we normalized the absolute change between the benchmark experiment and its replicate by the value of the benchmark experiment to obtain the relative change in percentage. As shown in Table S6, the mean relative change across all benchmarks was less than 1% for the total emission rate (excluding top three exhaled compounds) under ozone-free condition

and less than 1.5% for the total delta emission rate under ozone-present condition. For major skin ozonolysis products, the overall yield change was mostly below or around 10%. These results indicate good reproducibility of our experiments (experiment vs. replicate). Similarly, we calculated the absolute change for the listed parameters in Table S6 between no-shower experiments and benchmark experiments (Exp.1 and Exp.5), which was then normalized by the benchmark value correspondingly. Thus, we obtained a relative change for each no-shower experiment, to be compared with the relative change across all benchmark experiments.

Table S1. Details of the experimental conditions (Exp. No. 2 was an experiment with higher temperature, which was not included in the study).

Exp. No.	Steady-state temperature (°C)		Steady-state relative humidity (%)		Steady-state ozone level (ppb)		Notes
	Morning	Afternoon	Morning	Afternoon	Morning	Afternoon	
1	24.8	25.1	30	27	< 1	40	Benchmark (Day 1)
3	25.9	26.0	26	24	< 1	38	One-day no shower (Day 2)
4	25.6	25.6	25	24	< 1	35	Two-day no shower (Day 3)
5	25.1	25.5	25	25	< 1	42	Benchmark replicate (Day 4)
6	24.9	25.5	28	26	< 1	36	Lotion on skin (Day 7)

Table S2. Ingredient table of the lotion and the amount used in this study

**Lotion type**	**Ingredients**			
Basic hydrating lotion	Aqua, Glycerin, Brassica Campestris Seed Oil, Butyrospermum Parkii Butter, Ceteareth-12, Ceteareth-20, Cetearyl Alcohol, Ethylhexyl Stearate, Simmondsia Chinensis Seed Oil, Tocopherol, Caprylyl Glycol, Citric Acid, Sodium Hydroxide, Acrylates/C10-30 Alkyl Acrylate Crosspolymer, Sodium Gluconate, Phenoxyethanol			
	**Amount of lotion applied (g)**			
	Volunteer1	Volunteer 2	Volunteer 3	Volunteer 4
Morning	7.4	7.4	2.0*	6.8*
Afternoon	7.4	7.4	6.8*	2.0*

*The target amount of the lotion was 7.4 g. Due to mistakes during the experimental procedure, two volunteers applied different amounts.

Table S3. Difference (%) between steady-state values derived from fitting using sigmoidal Gompertz function and from the average values of last 15-minute measurements over the ozone-present period for species significantly correlating with 4-OPA ($R^2 \geq 0.90$, $p \leq 0.01$). Numbers in red indicate that fitting results are lower than last-15-min steady-state values.

m/z **(H^+)**	**Assigned Formula**	**Experiment No.**				
		**1**	**3**	**4**	**5**	**6**
45.0335	$C_2H_4OH^+$	1.4%	2.4%	3.4%	2.1%	-
41.0386	$C_3H_4H^+$	4.2%	-	-	-	-
43.0178	$C_2H_2OH^+$	2.9%	2.6%	3.5%	3.1%	1.9%
55.0542	$C_4H_6H^+$	1.7%	2.3%	1.9%	1.1%	1.0%
57.0699	$C_3H_4OH^+$	3.2%	6.6%	5.6%	4.1%	3.1%
57.0335	$C_4H_8H^+$	3.1%	2.5%	2.2%	2.4%	5.4%
61.0284	$C_2H_4O_2H^+$	1.4%	2.2%	4.6%	4.9%	3.1%
67.0542	$C_5H_6H^+$	3.5%	1.2%	1.5%	1.3%	0.5%
69.0335	$C_4H_4OH^+$	2.2%	2.8%	2.1%	0.9%	-
71.0491	$C_4H_6OH^+$	3.0%	2.2%	2.9%	2.4%	1.9%
73.0284	$C_3H_4O_2H^+$	4.0%	3.9%	3.1%	3.7%	4.4%
73.0648	$C_4H_8OH^+$	-	2.6%	2.9%	1.9%	-
75.0441	$C_3H_6O_2H^+$	3.8%	3.0%	2.6%	4.4%	-
81.0699	$C_6H_8H^+$	0.9%	1.1%	1.6%	1.1%	-0.01%
83.0855	$C_6H_{10}H^+$	0.6%	1.3%	1.4%	0.4%	-0.01%
87.0441	$C_4H_6O_2H^+$	3.4%	3.3%	3.4%	3.6%	3.0%
87.0804	$C_5H_{10}OH^+$	-	-	2.0%	-	-
95.0855	$C_7H_{10}H^+$	1.3%	3.1%	3.0%	-	-
97.1012	$C_7H_{12}H^+$	-0.1%	1.7%	2.3%	1.1%	0.6%
99.0441	$C_5H_6O_2H^+$	14.0%	19.5%	21.5%	21.2%	21.2%
101.0597	$C_5H_8O_2H^+$	1.7%	1.7%	2.1%	2.4%	2.7%
111.1168	$C_8H_{14}H^+$	1.0%	0.7%	-0.4%	-0.5%	-0.1%
121.0648	$C_8H_8OH^+$	-	-	2.1%	0.6%	-
123.1168	$C_9H_{14}H^+$	1.5%	2.1%	0.4%	0.1%	-0.2%
125.0961	$C_8H_{12}OH^+$	1.3%	0.4%	-0.1%	-2.0%	0.2%
125.1325	$C_9H_{16}H^+$	1.3%	1.2%	-	1.0%	-0.4%
141.1274	$C_9H_{16}OH^+$	-	-	-	-	-0.3%
143.1067	$C_8H_{14}O_2H^+$	-	-	-	-	-0.4%
143.1430	$C_9H_{18}OH^+$	1.5%	1.9%	1.8%	1.3%	-0.7%
145.1223	$C_8H_{16}O_2H^+$	-	-	-	-	6.1%
157.1587	$C_{10}H_{20}OH^+$	1.6%	2.3%	3.4%	2.7%	1.1%
171.1743	$C_{11}H_{22}OH^+$	2.9%	3.9%	4.3%	5.2%	4.0%

Table S4. Compounds calibrated to standard gas cylinders with average limit of detection (LOD) and total uncertainty together with standard deviation (std).

	***m/z* (H^+)**	**Compounds**	**LOD ± std (ppt)**	**Total uncertainty ± std (%)**
**PTR-ToF-MS**	33.0335	Methanol	229 ± 56	20 ± 6
	42.0338	Acetonitrile	24 ± 5	9 ± 3
	45.0335	Acetaldehyde	69 ± 8	6 ± 0.2
	47.0491	Ethanol	549 ± 300	23 ± 5
	54.0338	Acrylonitrile	9 ± 3	6 ± 0.5
	59.0491	Acetone	74 ± 19	7 ± 1
	63.0263	Dimethyl sulfide (DMS)	19 ± 7	5 ± 0.3
	69.0699	Isoprene	48 ± 22	6 ± 0.3
	71.0491	Methyl vinyl ketone (MVK)	21 ± 10	6 ± 1
	73.0648	Methyl ethyl ketone (MEK)	17 ± 4	6 ± 1
	79.0542	Benzene	10 ± 2	6 ± 0.4
	107.0855	Xylene	12 ± 5	5 ± 0.4
	121.1011	Trimethylbenzene	9 ± 5	5 ± 0.2
	137.1325	Alpha Pinene	11 ± 2	6 ± 0.2
	205.1951	Caryophyllene	35 ± 16	11 ± 3
	297.1951	Octamethylcyclotetrasiloxane (D4)	7 ± 6	6 ± 0.4
	371.3180	Decamethylcyclopentasiloxane (D5)	8 ± 4	5 ± 0.3
	127.1117	6-MHO	22	7
	101.0597	4-OPA	44	7
**Fast-GC**		Isoprene	4 ± 2	9 ± 1
		Propanal	15 ± 7	19 ± 9
		MVK	34 ± 19	13 ± 6
		Methacrolein	7 ± 3	16 ± 6

Table S5. Main O_3-reactive compounds[6] included in Eq. S1 for calculating the first-order ozone removal rate coefficient by occupants ($k_{occupants}$): their rate coefficient with ozone ($k_{i(O_3)}$), and steady-state mixing ratios during the hygiene experiments.

Compounds	$k_{i(O_3)}$* ppb^{-1}h^{-1}	Benchmark (Day1), ppb	One-day no shower (Day2) ppb	Two-day no shower (Day3) ppb	Benchmark replicate (Day4) ppb
**GA**	7.52×10^{-2}	1.03	1.21	1.47	0.66
**6-MHO**	3.44×10^{-2}	7.01	8.24	9.31	6.15
**4-MON**	3.76×10^{-2}	0.36	0.38	0.44	0.22
**4-MOD**	3.76×10^{-2}	0.21	0.22	0.23	0.16
**Isoprene**	1.12×10^{-3}	3.23	2.98	3.34	3.73
**MVK**	4.55×10^{-4}	0.62	0.53	0.31	0.35
**MACR**	1.05×10^{-4}	0.13	0.13	0.14	0.22
**2-nonenal**	1.14×10^{-3}	1.56	1.20	1.03	1.42
**Limonene**	1.92×10^{-2}	0.16	0.16	0.17	0.13

*The rate coefficient was taken from Table S1 in Zannoni et al.[6] and converted to the unit of ppb^{-1} h^{-1}. For MVK (methyl vinyl ketone) and MACR (methacrolein), as an averaged number was used in Zannoni et al.[6], values were taken from preferred numbers from IUPAC[9].

Table S6. Mean change in percentage for benchmark experiments compared to their replicate experiments (including Exp. 1 on Day 1 and Exp. 5 on Day 4 in this study and four pairs of replicate benchmark experiments from the previous ICHEAR study[8]), and for no-shower experiments (Exp. 3 on Day 2 and Exp. 4 on Day 3) compared to the benchmarks (Exp. 1 on Day 1 and Exp. 5 on Day 2) for various parameters reported in the study. Values in bold indicate that the mean relative change of no-shower experiments compared to the benchmarks for that parameter is significantly different from the mean relative change across overall benchmarks.

		Overall Benchmarks	One-day no shower	Two-day no shower
Ozone-free	total emission rate *	-0.7%	20.5%	**42.5%**
	acetic acid emission rate	0.7%	**84.4%**[#]	**171.8%**[#]
	$C_xH_yO_2$ emission rate (excluding acetic acid)	0.1%	**39.5%**[#]	**85.4%**[#]
Ozone-present	delta emission rate	1.2%	14.8%	**21.7%**
Overall ozone product yield for species in Table 1**	4-OPA	-1.0%	**14.8%**[#]	**27.5%**[#]
	6-MHO	-3.1%	**18.3%**	**33.4%**
	acetic acid	11.9%	4.8%	20.8%
	decanal	0.7%	0.4%	9.2%
	OH-6MHO	17.5%	8.3%	20.8%
	1,4-butanedial	3.2%	10.3%	**22.5%**
	acetaldehyde	12.5%	11.3%	7.6%
	hydroxy acetone /propionic acid	7.5%	0.3%	16.1%
	nonanal	2.5%	**-29.2%**	**-41.5%**
Squalene ozonolysis product surface yield	4-OPA	0.7%	**21.4%**[#]	**35.7%**[#]
	6-MHO	1.2%	16.3%	**29.2%**

*Breath compounds acetone, methanol and isoprene were subtracted from the total emission rate.
** Acetone was not shown here as it was also influenced by the breath emission.
[#] There is significant difference between the relative change of one-day no shower experiment and the relative change of two-days no shower experiment.

Table S7. Mass balance model sink terms for ozone removal during the hygiene experiments.

	Sinks		
	$k_{occupants}[O_3]_{ss}$ $(ppb\ h^{-1})$	$\lambda[O_3]_{ss}$ $(ppb\ h^{-1})$	$\sum_{gas-phase} k_{i(O_3)}[i]_{SS}[O_3]_{SS}$ $(ppb\ h^{-1})$
**Benchmark**	190	128	14
**One-day no shower**	199	122	15
**Two-day no shower**	199	112	16
**Benchmark replicate**	189	134	12

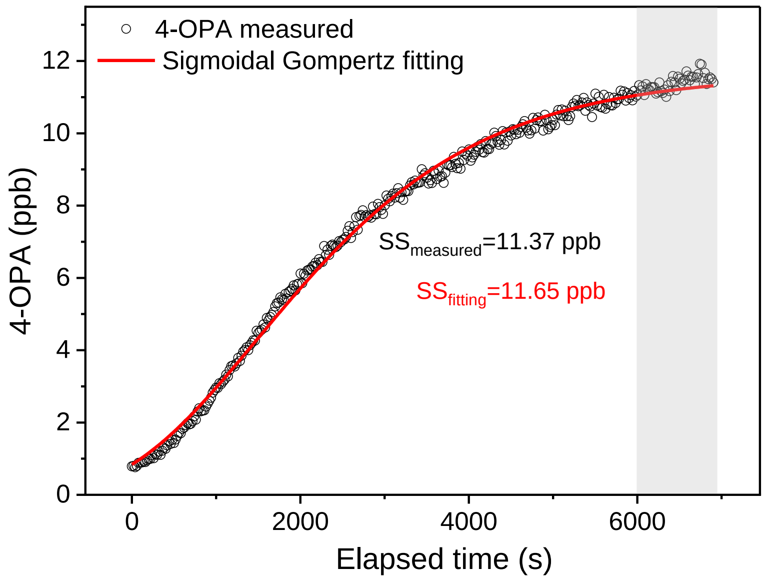

Figure S1. Sigmoidal Gompertz fitting example of 4-OPA during the afternoon session of Experiment No. 5 when ozone was present in the chamber (R^2 = 0.9964). Circles represent measured 4-OPA mixing ratio and red solid line represents the fitting curve. $SS_{measured}$ is the steady-state value averaged from measured data over the last 15 minutes before volunteers exited the chamber (shaded area). $SS_{fitting}$ is the steady-state value estimated from the fitting function.

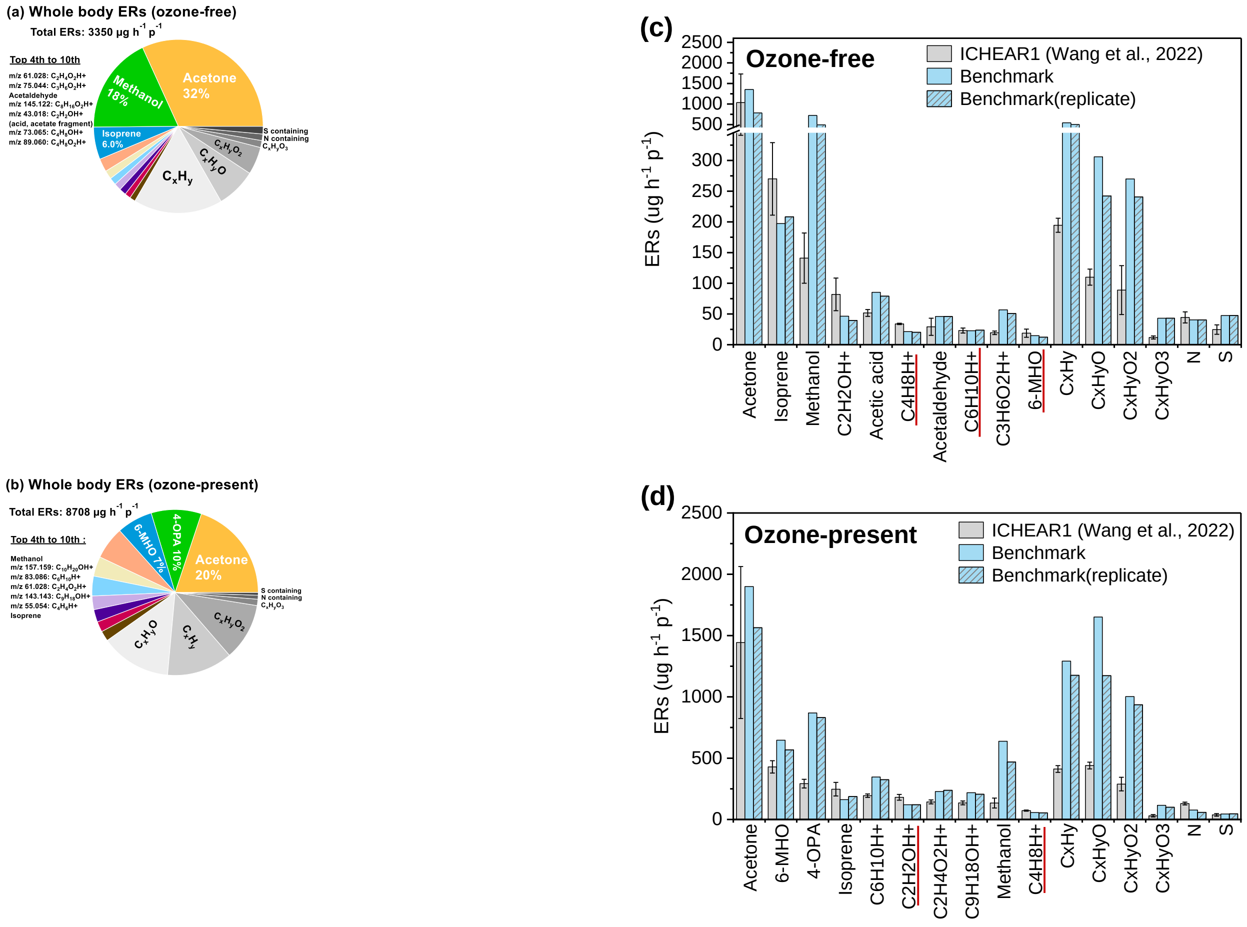

Figure S2. Total ERs and fractional contributions from the top 10 species and other grouped species based on the mean of the benchmark condition and its replicate (pie chart) under (a) ozone-free condition and (b) ozone-present condition. Whole-body emission rates (ERs) of top ten species identified during ICHEAR1 (Wang et al., 2022) and their corresponding ERs from the benchmark and its replicate in this study (bar chart) under (c) ozone-free condition and (d) ozone-present condition. Red underlines in (c) and (d) refer to the species which were not ranked as top 10 in this study.

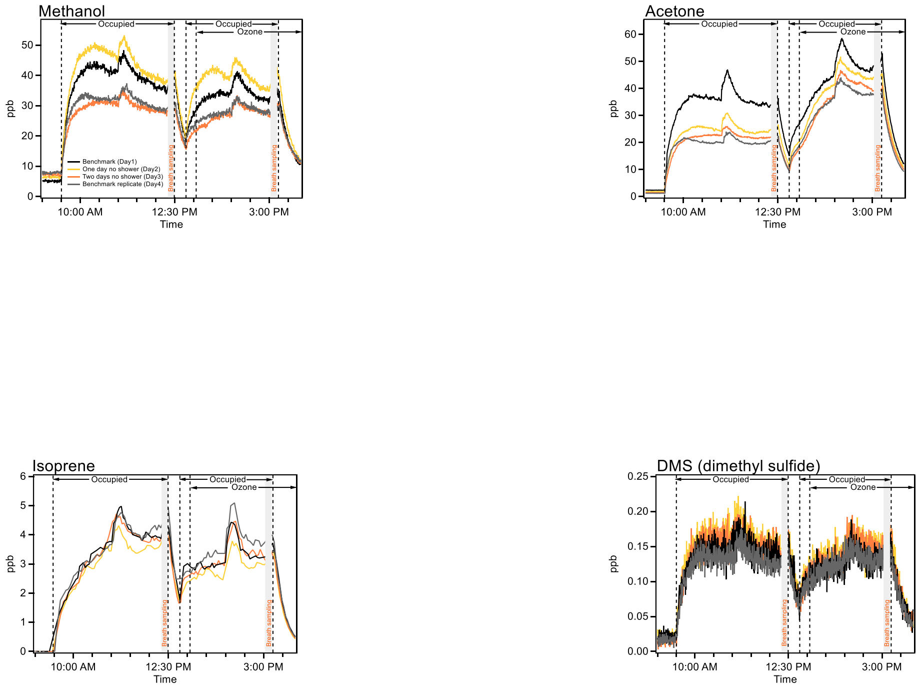

Figure S3. Time series of selected major breath-borne VOCs measured during the experiments with different hygiene levels. The shaded gaps represent the time period where PTR-MS measured breath.

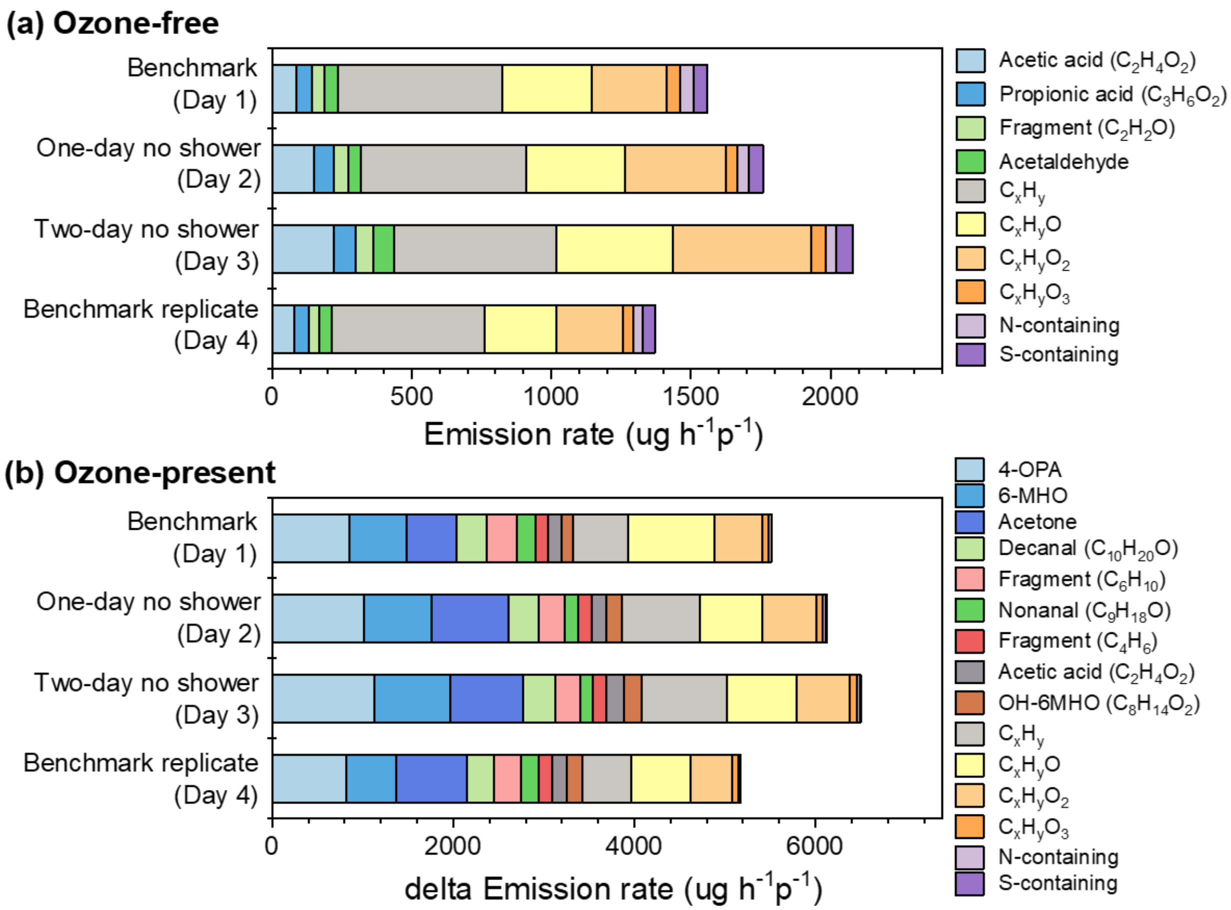

Figure S4. (a) Total emission rates excluding the top three species (breath compounds: acetone, methanol and isoprene) and fractional contributions from other strongly emitted species and the rest of the species grouped into six chemical family groups during the hygiene experiments under ozone-free condition. (b) Delta emission rates (the difference between the ER during steady state with ozone present and absent) and fractional contributions from the strongly emitted species (species that appear among the top 10 contributors in all four experiments) and from rest of species grouped into six groups during the hygiene experiments under ozone-present condition.

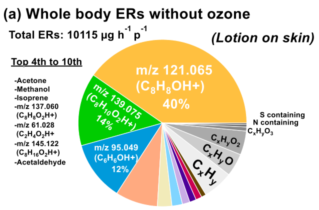

Figure S5. Total whole-body ERs and fractional contributions from top 10 species and other grouped species during the experiment with lotion on skin under (a) ozone-free condition and (b) ozone-present condition.

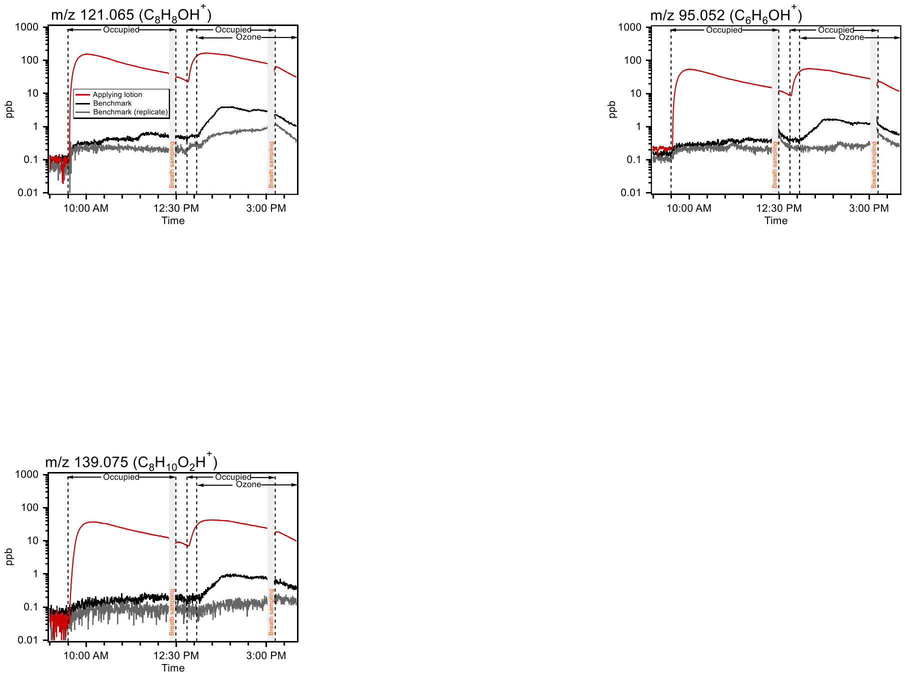

Figure S6. Time series of species for which the lotion was a strong source during the benchmark experiment, its replicate and the lotion-on-skin experiment. The presented species were the most abundant VOCs observed in the lotion-only experiment, showing no clear impact of ozone. The shaded gaps represent the time periods where PTR-MS measured breath.

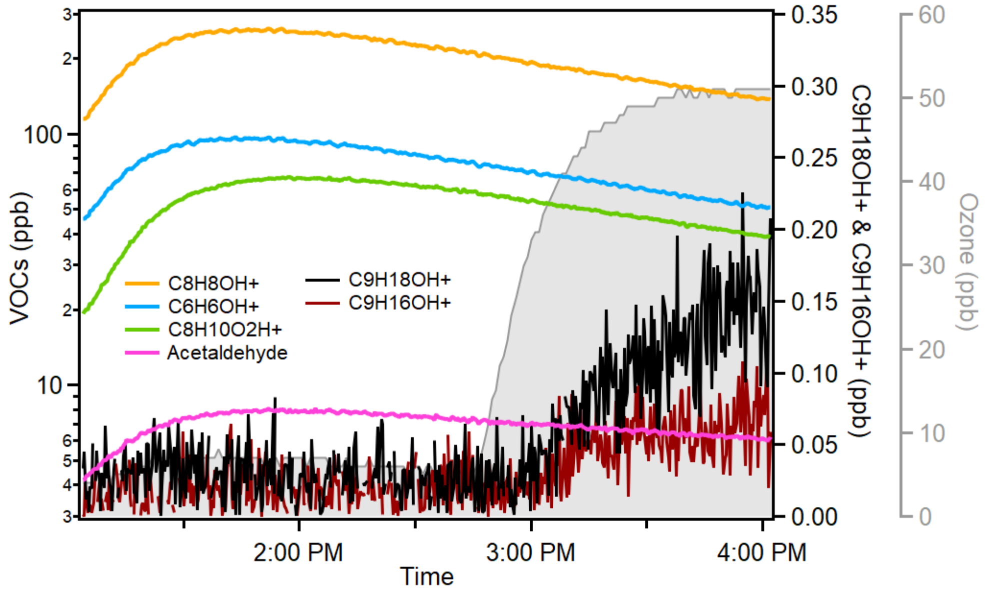

Figure S7. Time series of acetaldehyde, major species originating from the lotion and potential lotion-ozone reaction products ($C_9H_{18}O$ and $C_9H_{16}O$) together with ozone mixing ratios in the chamber during lotion-only experiment.

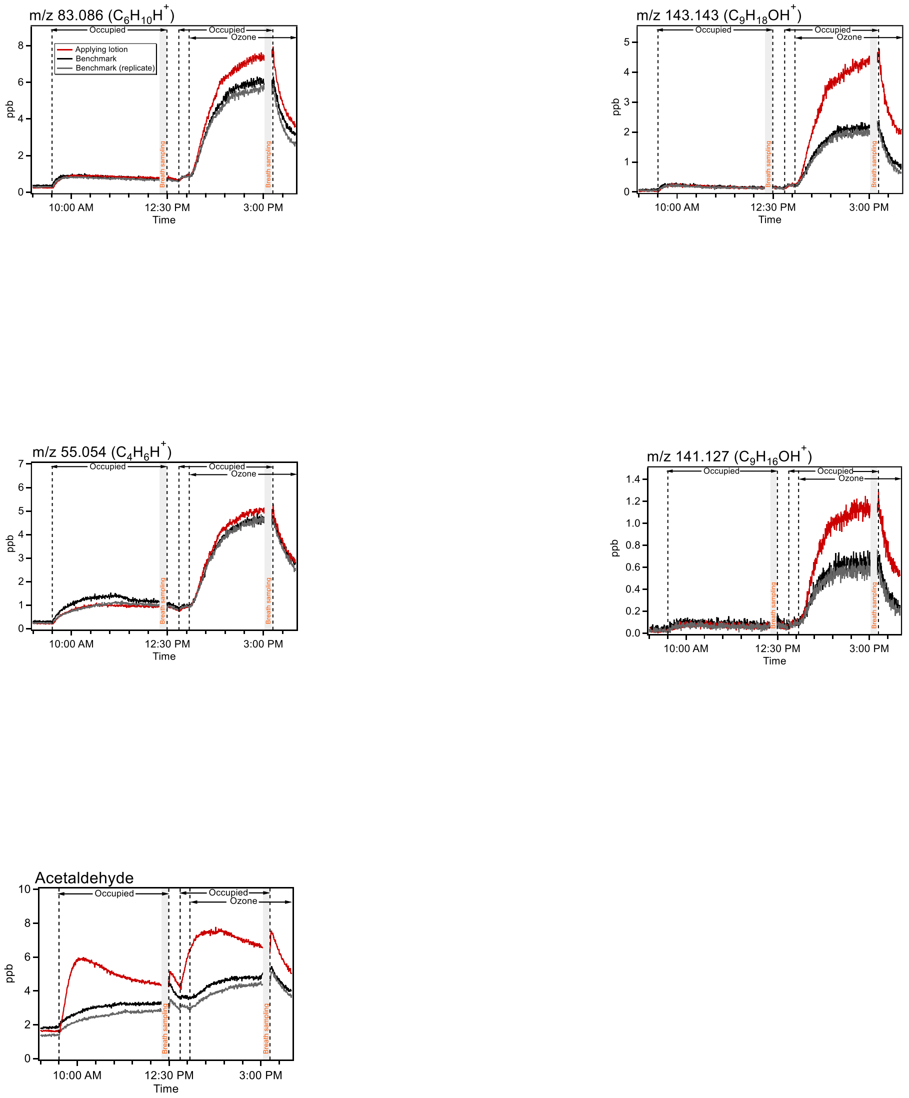

Figure S8. Time series of species in Figure 4b showing higher ozone reaction product yields when lotion was applied on skin compared to the no-lotion condition (benchmarks from hygiene experiments).

References

(1) Lindinger, W.; Hansel, A.; Jordan, A. On-Line Monitoring of Volatile Organic Compounds at Pptv Levels by Means of Proton-Transfer-Reaction Mass Spectrometry (PTR-MS) Medical Applications, Food Control and Environmental Research. *International Journal of Mass Spectrometry and Ion Processes* **1998**, *173* (3), 191–241.

(2) Cappellin, L.; Karl, T.; Probst, M.; Ismailova, O.; Winkler, P. M.; Soukoulis, C.; Aprea, E.; Mark, T. D.; Gasperi, F.; Biasioli, F. On Quantitative Determination of Volatile Organic Compound Concentrations Using Proton Transfer Reaction Time-of-Flight Mass Spectrometry. *Environ Sci Technol* **2012**, *46* (4), 2283–2290. https://doi.org/10.1021/es203985t.

(3) de Gouw, J.; Warneke, C. Measurements of Volatile Organic Compounds in the Earth's Atmosphere Using Proton-transfer-reaction Mass Spectrometry. *Mass spectrometry reviews* **2007**, *26* (2), 223–257.

(4) Zhao, J.; Zhang, R. Proton Transfer Reaction Rate Constants between Hydronium Ion (H3O+) and Volatile Organic Compounds. *Atmospheric Environment* **2004**, *38* (14), 2177–2185. https://doi.org/10.1016/j.atmosenv.2004.01.019.

(5) Wisthaler, A.; Weschler, C. J. Reactions of Ozone with Human Skin Lipids: Sources of Carbonyls, Dicarbonyls, and Hydroxycarbonyls in Indoor Air. *Proceedings of the National Academy of Sciences* **2010**, *107* (15), 6568–6575.

(6) Zannoni, N.; Lakey, P. S. J.; Won, Y.; Shiraiwa, M.; Rim, D.; Weschler, C. J.; Wang, N.; Ernle, L.; Li, M.; Bekö, G.; Wargocki, P.; Williams, J. The Human Oxidation Field. *Science* **2022**, *377* (6610), 1071–1077. https://doi.org/doi:10.1126/science.abn0340.

(7) Bekö, G.; Wargocki, P.; Wang, N.; Li, M.; Weschler, C. J.; Morrison, G.; Langer, S.; Ernle, L.; Licina, D.; Yang, S.; Zannoni, N.; Williams, J. The Indoor Chemical Human Emissions and Reactivity (ICHEAR) Project: Overview of Experimental Methodology and Preliminary Results. *Indoor air* **2020**, *30* (6), 1213–1228. https://doi.org/10.1111/ina.12687.

(8) Wang, N.; Ernle, L.; Beko, G.; Wargocki, P.; Williams, J. Emission Rates of Volatile Organic Compounds from Humans. *Environ Sci Technol* **2022**, *56* (8), 4838–4848. https://doi.org/10.1021/acs.est.1c08764.

(9) *IUPAC – Task Group on Atmospheric Chemical Kinetic Data Evaluation*. https://iupac.aeris-data.fr/ (accessed 2023-06-20).
